# Improve the efficiency and accuracy of ophthalmologists’ clinical decision-making based on AI technology

**DOI:** 10.1186/s12911-024-02587-z

**Published:** 2024-07-09

**Authors:** Yingxuan Guo, Changke Huang, Yaying Sheng, Wenjie Zhang, Xin Ye, Hengli Lian, Jiahao Xu, Yiqi Chen

**Affiliations:** 1 School of Ophthalmology and Optometry, Eye Hospital, Wenzhou Medical University, Wenzhou, Zhejiang China; 2 Center for Rehabilitation Medicine, Department of Ophthalmology, Zhejiang Provincial People’s Hospital (Affiliated People’s Hospital, Hangzhou Medical College), Hangzhou, Zhejiang China

**Keywords:** Electronic medical records, Natural language processing, Named entity recognition, Diagnostic prediction, Diagnosis accuracy, Fundus diseases

## Abstract

**Background:**

As global aging intensifies, the prevalence of ocular fundus diseases continues to rise. In China, the tense doctor-patient ratio poses numerous challenges for the early diagnosis and treatment of ocular fundus diseases. To reduce the high risk of missed or misdiagnosed cases, avoid irreversible visual impairment for patients, and ensure good visual prognosis for patients with ocular fundus diseases, it is particularly important to enhance the growth and diagnostic capabilities of junior doctors. This study aims to leverage the value of electronic medical record data to developing a diagnostic intelligent decision support platform. This platform aims to assist junior doctors in diagnosing ocular fundus diseases quickly and accurately, expedite their professional growth, and prevent delays in patient treatment. An empirical evaluation will assess the platform’s effectiveness in enhancing doctors’ diagnostic efficiency and accuracy.

**Methods:**

In this study, eight Chinese Named Entity Recognition (NER) models were compared, and the SoftLexicon-Glove-Word2vec model, achieving a high F1 score of 93.02%, was selected as the optimal recognition tool. This model was then used to extract key information from electronic medical records (EMRs) and generate feature variables based on diagnostic rule templates. Subsequently, an XGBoost algorithm was employed to construct an intelligent decision support platform for diagnosing ocular fundus diseases. The effectiveness of the platform in improving diagnostic efficiency and accuracy was evaluated through a controlled experiment comparing experienced and junior doctors.

**Results:**

The use of the diagnostic intelligent decision support platform resulted in significant improvements in both diagnostic efficiency and accuracy for both experienced and junior doctors (*P* < 0.05). Notably, the gap in diagnostic speed and precision between junior doctors and experienced doctors narrowed considerably when the platform was used. Although the platform also provided some benefits to experienced doctors, the improvement was less pronounced compared to junior doctors.

**Conclusion:**

The diagnostic intelligent decision support platform established in this study, based on the XGBoost algorithm and NER, effectively enhances the diagnostic efficiency and accuracy of junior doctors in ocular fundus diseases. This has significant implications for optimizing clinical diagnosis and treatment.

## Introduction

As the aging of the population continues to increase, the incidence of eye diseases is also rising year by year. Common retinal diseases include macular depression, vitreous hemorrhage, macular hole, retinal detachment, and diabetic retinopathy. Among them, macular diseases such as epiretinal membrane (ERM) and macular hole are one of the important causes of visual impairment, causing visual impairment or even blindness in developed and developing countries [1]. Early diagnosis and timely treatment are crucial for achieving the best visual outcomes [2–4]. Therefore, it is very necessary to detect and follow up in time for early retinal disease.

In the clinical diagnosis and treatment of ocular fundus diseases, the ability to make rapid and accurate diagnoses is crucial. Missed or misdiagnosed cases during this process can not only cause irreversible damage to patients but also severely test the quality of medical services. In China, due to the exceptionally low doctor-patient ratio, this challenge is particularly prominent for ophthalmologists. They must undertake a significant amount of analysis and judgment day after day, testing not only their professional knowledge but also their work efficiency. Against this backdrop, improving the speed and accuracy of diagnoses can not only improve patient outcomes but also significantly enhance overall medical quality [5].

Clinical Decision Support Systems (CDSS) are computer programs designed to assist in the diagnosis of diseases, and their importance has become increasingly prominent with the growth of applications of mathematical sciences, engineering principles, and computer technology in the medical field. Especially in the diagnosis and treatment of ocular fundus lesions, the use of artificial intelligence (AI) applications such as optical coherence tomography (OCT) and intelligent image processing techniques has become a key tool for effectively improving diagnostic accuracy and efficiency [6–10]. For example, the CDSS developed by Vellakani S et al. based on OCT technology can assist ophthalmologists in more accurately detecting and classifying eye diseases [11], while the cloud-based CDSS developed by Tanya S M et al. facilitates remote diagnosis and treatment, demonstrating the wide range of applications and potential of CDSS in ophthalmology [5]. However, despite significant progress in AI technology for image analysis, the value of large volumes of medical record text data is also worth exploring. Fully utilizing the textual information from previous cases as a resource for auxiliary diagnosis to explore its feasibility in improving the comprehensiveness, accuracy, and efficiency of early diagnosis of ocular fundus diseases [12–14].

In recent years, traditional paper medical records have been largely replaced by electronic medical records (EMR), which contain basic information about patients’ diseases, such as chief complaints, current medical history, past medical history, and examination results [15]. However, in the early stages of EMR development, due to the lack of unified standards and norms, a large amount of unstructured free text was used, hindering the effective use of EMR data [16, 17]. The same issue exists in ophthalmic EMR, which contains a significant amount of specialized ophthalmic vocabulary with detailed and specific descriptions of the eye. Therefore, when recognizing EMR, it is necessary to consider both general descriptive entities in the medical records, such as body parts, symptoms, signs, and examination results, as well as a large number of accurate ophthalmic terminologies, such as macula, retina, visual acuity, and axial length. We attempted to utilize artificial intelligence Named Entity Recognition (NER) technology to effectively extract EMR data rich in ophthalmic terminologies.

The Extreme Gradient Boosting (XGBoost) algorithm is an ensemble classifier based on gradient boosting trees proposed by Tianqi Chen [18]. It has good fitting ability for data and can directly control the degree of overfitting by incorporating regularization terms into the objective function. Due to its open-source code, it has important applications in many machine learning and data mining tasks [19–22]. We used this algorithm to construct an ophthalmic diagnostic intelligent decision support platform.

In this study, the structured processing of free text is the first and crucial step in disease diagnosis decision-making. This article provides a detailed introduction to the NER model selection experiment. Based on the optimized model, we constructed an ophthalmic diagnostic intelligent decision support platform using the XGBoost machine learning algorithm and explored its feasibility in improving the efficiency and accuracy of clinical ophthalmologists in diagnosing ocular fundus diseases.

## Materials and methods

### System development technical roadmap

**Fig. 1:**
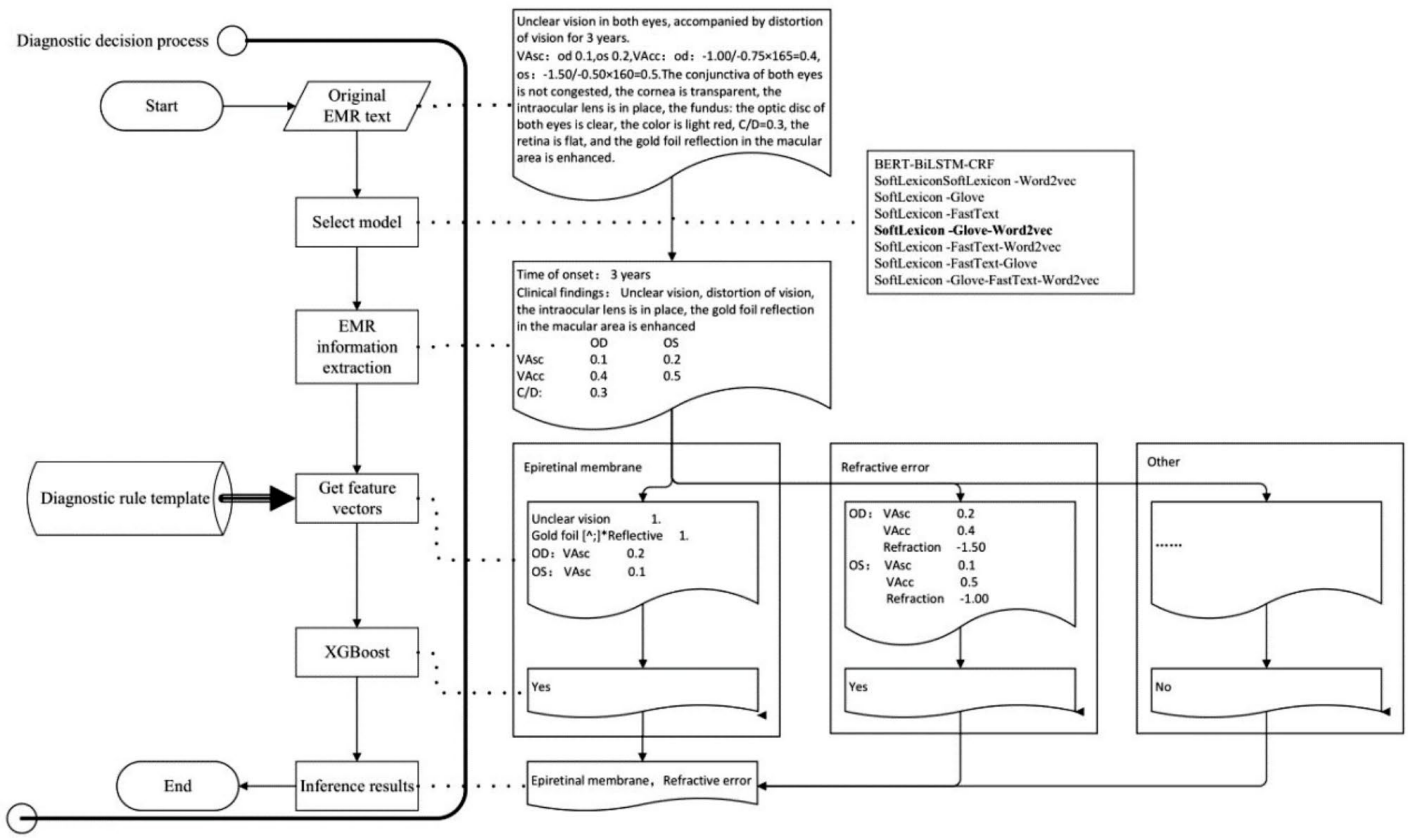
Technology roadmap

The construction of the ophthalmic diagnostic intelligent decision support platform required the support of AI technology, first of which was Natural Language Processing (NLP) technology. This involved training NER models using historical medical records, selecting the optimal model, and extracting important diagnostic information from the patient’s EMR. Then, according to the diagnostic rule template, various disease feature vectors were generated, and the XGBoost algorithm of machine learning technology was used to infer the optimal disease diagnosis. The input of XGBoost is the vector of key diagnostic feature words extracted from ophthalmic EMRs, and the output is the recommended ophthalmic disease name for diagnosis. The specific technical roadmap was shown in Fig. 1.

### Determination of the best NER model

There are many models related to NER, including commonly used models such as Bidirectional Encoder Representations from Transformers (BERT) [23], Long Short Term Memory (LSTM) [24], and conditional random field (CRF) [25]. To determine the most suitable NER model for ophthalmic electronic medical records, we use the SoftLexicon model proposed by ALC 2020 [26] as the text classification model. This model incorporates dictionary information at the Chinese character level NER model, resulting in good NER performance. By combining different word vector models for effect comparison, we aim to determine the best named entity recognition model for ophthalmic electronic medical record recognition scenarios.

The sample and test set data came from the EMR desensitization database of a tertiary grade-A eye hospital in Zhejiang Province. The time period was from January 2022 to December 2022. Since deep learning-based methods required high-quality labeled data, after the EMRs were cleaned, they were labeled by a professional team of ophthalmic clinical doctors, and the labeled tags were set according to clinically common diagnostic and treatment basis, including chief complaints, history of present illness, past history, systemic disease history, clinical examinations (specialized and supplementary examination) and operative procedures etc. The annotated tags were shown in Table 1. The experiment used 2280 standardized EMRs annotated by a team of ophthalmologists, with 1824 randomly selected as the training set and the remaining 456 as the test set.

**Table 1:** Label list

Main label	Sub label
Chief complaint	
History of present illness	
Past history	
Systemic disease history	
Special examination	Uncorrected vision
	Corrected vision
	Intraocular pressure
	Ophthalmic examination
Supplementary examination	B-ultrasound
	Axial length
	Corneal endothelioscope
	Optical coherence tomography (OCT)
Disease and diagnosis	
Operative procedures	

In model selection experiment, the Chinese classical NER BERT-BiLSTM-CRF model was used as the baseline model [27, 28], and the SoftLexicon model combined with the word vector Glove, Word2vec and FastText was used as the test model [29, 30]. The evaluation metrics were Precision, Recall, and F1 score, F1 is the harmonic mean of Precision and Recall, which is used to measure the accuracy and recall of the model in general. The formula for calculation is:

1 $$\text{F}1=2\text{*}\frac{\text{P}\text{r}\text{e}\text{c}\text{i}\text{s}\text{i}\text{o}\text{n}\text{*}\text{R}\text{e}\text{c}\text{a}\text{l}\text{l}}{\text{P}\text{r}\text{e}\text{c}\text{i}\text{s}\text{i}\text{o}\text{n}+\text{R}\text{e}\text{c}\text{a}\text{l}\text{l}}$$

After comparing different word vectors, the optimal results are shown in Table 2. The computational power and performance of the optimal model and the baseline model were evaluated by the time and GPU days consumed during a single round of training.

**Table 2:** Experimental results of different models

Model	Precision(%)	Recall(%)	F1(%)
BERT-BiLSTM-CRF	90.81	92.98	91.88
SoftLexicon	91.80	93.40	92.59
SoftLexicon -Word2vec	92.00	93.06	92.53
SoftLexicon -Glove	92.02	93.37	92.69
SoftLexicon -FastText	91.76	93.26	92.50
**SoftLexicon -Glove-Word2vec**	**92.28**	**93.76**	**93.02**
SoftLexicon -FastText-Word2vec	92.41	93.37	92.89
SoftLexicon -FastText-Glove	91.08	93.68	92.73
SoftLexicon -Glove-FastText-Word2vec	92.08	93.73	92.90

The SoftLexicon-Love-Word2vec model with the highest F1 score had a shorter average training times in different batches than the baseline model BERT-BiLSTM-CRF. When inferring 1000 text samples, the SoftLexicon-Glove-Word2vec model took 7.94 s, which was 7.69% faster than the baseline model’s 8.55 s. The search time for the SoftLexicon-Glove-Word2vec model on a 2080Ti GPU was 0.21 GPU days, which was shorter than the baseline model’s 0.27 GPU days on a 2080Ti GPU.

Based on the above experimental results, the SoftLexicon-Glove-Word2vec model was the best model for this study. We used this model to build an ophthalmic diagnostic intelligent decision support platform for fundus diseases.

### Development of the diagnostic intelligent decision support platform

Through research and communication with clinical ophthalmologists and based on medical diagnosis practices, we have found that the diagnosis methods for ophthalmology diseases in EMRs generally have distinct distribution characteristics of key feature words. For example, for vitreous hemorrhage, the special examination results will clearly appear with a description containing the term “hemorrhage”. For refractive errors, the chief complaint generally appears with the term “blurred vision” and the supplementary examination results show an uncorrected vision less than 1.0. The diagnostic rule templates for ocular fundus diseases are constructed by analyzing the patterns and relationships among various features extracted from EMRs, such as patient demographics, chief complaint, medical history, and examination results. By identifying significant correlations between these features and specific ocular fundus diseases, we can create rules for disease diagnosis. These templates provide junior doctors with a structured approach to diagnosing ocular fundus diseases.

After extracting feature information from free text EMRs. Based on the diagnostic rule template, generate feature vectors, and then use XGBoost classifier to infer the corresponding disease diagnosis. Using the training set and test set of the above samples, the predictive performance of the XGBoost classifier on the test set is shown in Table 3.

**Table 3:** Prediction results of different diseases in the XGBoost model

Diagnosis	Precision(%)	Recall(%)	F1(%)
Silicon oil-filled eye	0.928	0.983	0.962
Pathological myopia	0.885	0.935	0.909
Vitreous hemorrhage	0.913	0.87	0.891
Cataract	0.884	0.84	0.861
Retinal detachment	0.805	0.892	0.846
Epiretinal membrane	0.887	0.795	0.839
Diabetic retinopathy	0.796	0.807	0.801
Macular hole	0.891	0.719	0.796
Refractive error	0.781	0.805	0.793

**Fig. 2:**
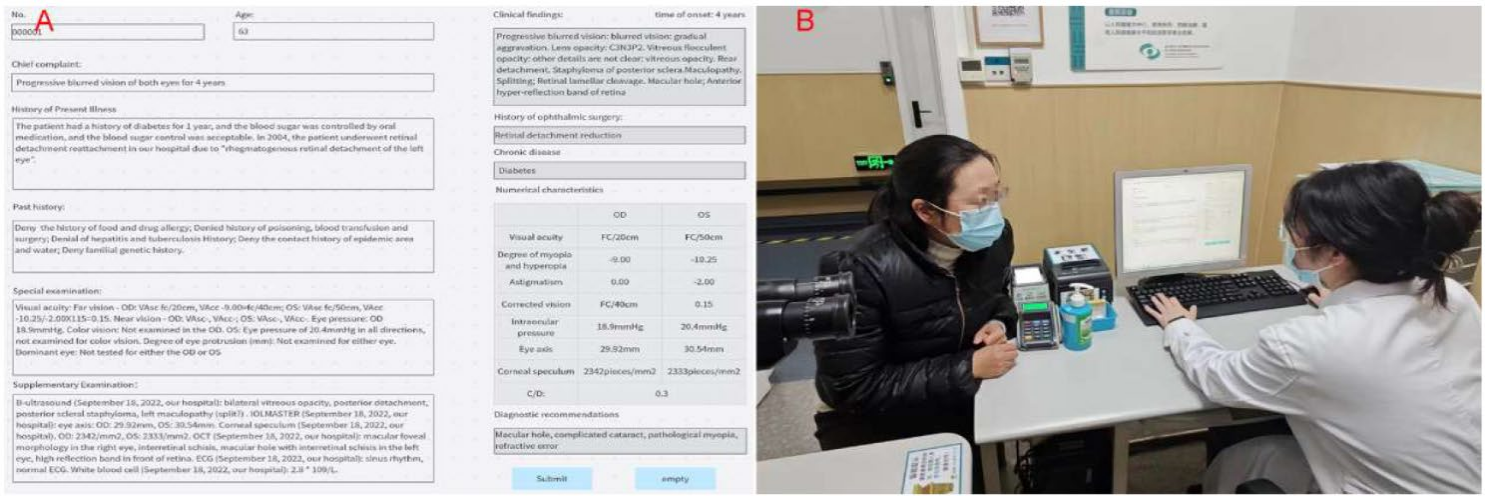
(**A**): Diagnostic inference interface. (**B**): The scene of trial use in the clinic room (Consent for publication had been obtained from the doctor and patient in the picture)

By using NER and AI technology to structurally process EMRs, we could extract and utilize feature vectors. To improve the efficiency and accuracy of clinical ophthalmologists in diagnosing retinal diseases, we developed an ophthalmic diagnostic intelligent decision support platform in combination with the XGBoost algorithm. Figure 2 shows the functional interface of ophthalmic diagnostic intelligent decision support platform and the scenarios of its trial use in the clinic room. In Fig. 2.A, the left side is the input area for electronic medical record information, while the right side is the result display area. After clicking the “Submit” button, the clinical findings column on the right presented the meaningful key medical terms identified by NER for assist diagnosis. The patient’s history of ophthalmic surgery and chronic disease names could also be extracted simultaneously. The numerical feature column displays the ophthalmic examination numerical features related to the EMR. The recommended diagnosis column displays the recommended diagnosis disease names obtained through NER of the key medical terms in the EMR. Clinical doctors could use the recommended diagnosis to better perform diagnosis and treatment work, reduce missed diagnosis and misdiagnosis, and save patients’ visiting time.

## Application effect

### Evaluation metrics

We selected 60 EMRs of the five common eye diseases in the fundus in 2023, including retinal detachment, epiretinal membrane, vitreous hemorrhage, macular hole, and diabetic retinopathy. They were divided into two groups: the ophthalmic diagnostic intelligent decision support platform group and the non-diagnostic decision support group, with 30 cases in each group. The characteristics of population composition and the distribution of disease types are shown in Table 4, Help = 0 represented not using the diagnostic decision support platform, and Help = 1 represented using the diagnostic decision support platform. All 60 EMRs underwent strict three-level clinical quality control, with the first level completed by the clinical department, the second level completed by chief physicians and associate chief physicians, and the third level completed by the department heads, functional department heads, and chief physicians. This ensures the data quality and the accuracy of the disease diagnosis for each EMR.

**Table 4:** Basic information of EMR and disease composition

Characteristic	Groups	*N* (%)
Age(mean ± SD)	59.65 ± 11.07	60(100)
Gander	M	26(43.3)
	F	34(56.7)
Diagnosis		
Help = 0	Diabetes retinopathy	2(3.3)
	Macular hole	7(11.7)
	Vitreous hemorrhage	3(5.0)
	Epiretinal membrane	9(15.0)
	Retinal detachment	9(15.0)
Help = 1	Diabetes retinopathy	3(5.0)
	Macular hole	7(11.7)
	Vitreous hemorrhage	3(5.0)
	Epiretinal membrane	8(13.3)
	Retinal detachment	9(15.0)

Invited 6 clinical ophthalmologists, divided into two groups based on whether their clinical experience is greater than or equal to 10 years. Those with greater than or equal to 10 years of experience were in the senior doctor group, while those with less than 10 years of experience were in the junior doctor group, with 3 doctors in each group. They completed diagnostic experiments on different 30 EMRs of common clinical fundus diseases under two conditions: not using the ophthalmic diagnostic intelligent decision support platform, and using the ophthalmic diagnostic intelligent decision support platform. The time used for diagnosis and the name of the disease were recorded. The diagnosis time was defined as the time from when the EMR appears on the screen until the doctor selected the diagnosis and clicked the “save” button. The experimental program would automatically record the diagnosis time. The disease diagnosis in the EMR of third-level quality control was used as the gold standard for this experiment. All doctors participating in the experiment were considered to have hit the target if at least one of their three diagnoses was consistent with the gold standard, otherwise considered a miss. Using the R (version 4.1.0; R Studio 2021.09.0) statistical software for analysis and figures drawing, survival analysis method was used to analyze whether there were differences in diagnosis time and diagnostic accuracy of different doctor groups with and without using the ophthalmic diagnostic intelligent decision support platform. It was considered statistically significant difference when *p* < 0.05.

### Evaluation results

**Fig. 3:**
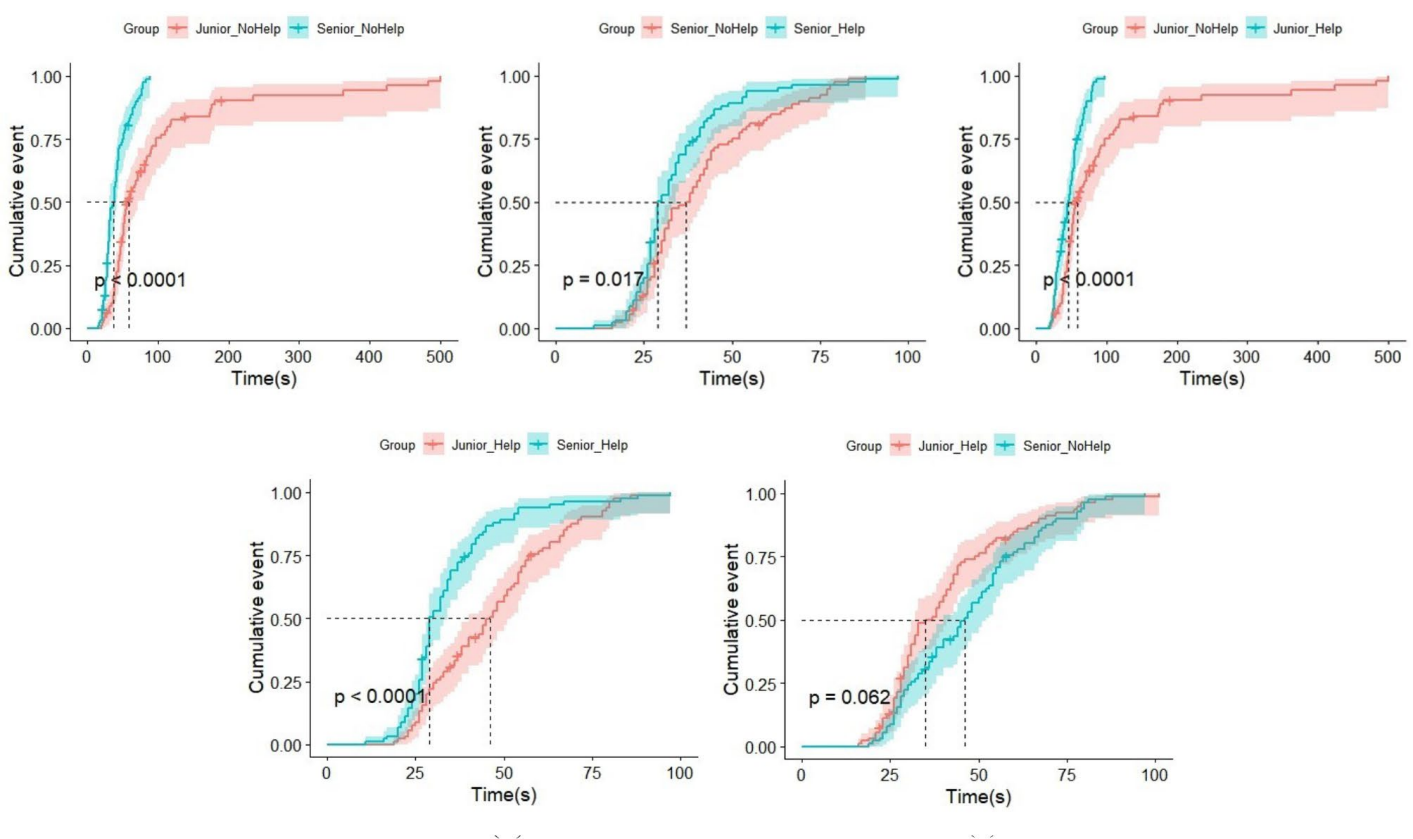
Survival analysis chart

The survival analysis statistical results were shown in Table 5. In the table, Group = 0 represented the junior doctor group, and Group = 1 represented the senior doctor group. Help = 0 represented not using the ophthalmic diagnostic intelligent decision support platform, and Help = 1 represented using the ophthalmic diagnostic intelligent decision support platform. Figure 3 shows the survival analysis curve under different conditions.

**Table 5:** Statistical analysis result table

Characteristics	Cases	Proportion	Correct	Median	Confidence interval		Log-rank	
		(%)	Cases	(s)	0.95LCL	0.95UCL	X^2^	*p*
**Junior group(0)**								
Help = 0	90	50	78	58	51	72	30.4	< 0.0001
Help = 1	90	50	86	46	40	52		
**Senior group(1)**								
Help = 0	90	50	84	37	32	42	5.7	0.02
Help = 1	90	50	87	29	29	34		
**Help = 0**								
Group = 0(Junior)	90	50	78	58	51	72	48.7	< 0.0001
Group = 1(Senior)	90	50	84	37	32	42		
**Help = 1**								
Group = 0(Junior)	90	50	86	46	40	52	20.4	< 0.0001
Group = 1(Senior)	90	50	87	29	29	34		
**Different variables**								
Group = 0, Help = 1	90	50	86	46	40	52	3.5	0.06
Group = 1, Help = 0	90	50	84	37	32	42		

## Result analysis and discussion

Clinical word, including chief complaint, current medical history, past history, systemic medical history, clinical examination, and imaging examination reports provided by examination doctors, is an important factor in the diagnosis of various components of electronic medical records. Based on the above clinical word, the ophthalmic diagnostic intelligent decision support platform has made diagnostic predictions. In the diagnostic test of five common fundus diseases, including retinal detachment, epiretinal membrane, vitreous hemorrhage, macular hole and diabetes retinopathy, the results and analysis are as follows.

Based on log-rank test, there was a statistically significant difference (*p* < 0.0001) in the accuracy of diagnosis of the five common fundus diseases between junior doctors when using or not using the aided diagnosis platform, with an improvement in accuracy, and the median time was 46 s, which was 12 s less than not using the platform. This indicated that the ophthalmic diagnostic intelligent decision support platform significantly improves the accuracy and speed of diagnostic decisions made by junior doctors.

Based on log-rank test, there was a statistically significant difference (*p* = 0.02) in the accuracy of diagnosis of the five common fundus diseases between senior doctors when using or not using the ophthalmic diagnostic intelligent decision support platform, with an improvement in accuracy, and the median time was 29 s, which was 8 s less than not using the platform. This indicated that the ophthalmic diagnostic intelligent decision support platform also had an auxiliary effect on the diagnostic decisions of senior doctors, but the effect was not as good as for junior doctors. This may have been due to the fact that the experience level of senior doctors was generally higher than that of junior doctors, thereby reducing the effectiveness of the ophthalmic diagnostic intelligent decision support platform.

There was a statistically significant difference (*p* < 0.0001) in the accuracy and efficiency of diagnosis between junior doctors and senior doctors when not using the ophthalmic diagnostic intelligent decision support platform, and the median time was 58 s and 37 s respectively. The chi-square test value was 48.7. This further indicated that senior doctors had significant advantages in terms of diagnostic speed and accuracy compared to junior doctors.

There was a statistically significant difference (*p* < 0.0001) in the accuracy and efficiency of diagnosis between junior doctors and senior doctors when using the ophthalmic diagnostic intelligent decision support platform, and the median time was 46 s and 29 s respectively. The chi-square test value was 20.4.

There was no statistically significant difference (*p* = 0.06) in the accuracy of diagnosis of common fundus diseases between junior doctors using the ophthalmic diagnostic intelligent decision support platform and senior doctors not using the platform, and the median time was 46 s and 37 s respectively. In combination with 3.2, it was shown that the gap in diagnostic speed and accuracy between junior doctors and senior doctors had decreased to some extent after the use of the ophthalmic diagnostic intelligent decision support platform by junior doctors.

NLP had a wide range of applications and is an interdisciplinary field that includes computer science, AI, and linguistics [31–33]. It had driven the development of AI text processing technologies, unleashing the value of non-structured text that had been accumulated for many years.

In this study, the SoftLexicon model was used to fuse different word vectors. The results showed ① The SoftLexicon-Glove-Word2vec model, which integrated Glove and Word2vec word vectors, obtained the best experimental results with an F1 score of 93.02. ② The SoftLexicon-Glove-Word2vec, SoftLexicon-FastText-Word2vec, and SoftLexicon-FastText-Glove models that fused two word vectors concatenate character, word, and bigram text feature vectors. They considered both global and local information, making the feature information more abundant, and the overall evaluation indicators were higher than those of the models that fused a single word vector. ③ The SoftLexicon-Glove-FastText-Word2vec model had a slightly lower F1 score than SoftLexicon-Glove-Word2vec, possibly due to the overabundance of fused features, which slowed down model training and inference speed, causing model overfitting and reducing performance on the test set. However, it still performed better than other models. ④ Models that fuse a single word vector had lower experimental results than models that fuse two or three word vectors. Based on the above analysis, the SoftLexicon-Glove-Word2vec model performed best in NER ophthalmic EMR.

## Limitations

This experiment is the first exploration of NER technology in the field of clinical ophthalmology, and only a few commonly used models have been studied. Although the data samples collected were labeled by a professional medical team, they still inevitably contain unmarked or incorrectly labeled entities, which may not be recognized or incorrectly recognized. In addition, the sample collection is single-center data, and ENRs may have template writing habits, which may affect the recognition results of this experiment to some extent. Finally, the XGBoost classifier algorithm may also affect the results of disease diagnosis reasoning.

## Conclusions

Based on the comprehensive analysis, this study found that the AI NLP NER technology based on fused medical vocabulary information had a good effect on NER of Chinese EMR information in ophthalmology. By extracting the medical vocabulary features, the eye disease aided diagnosis platform constructed could make disease diagnosis predictions that could improve the clinical decision-making ability of junior doctors, narrow the clinical diagnostic gap with senior doctors, and improve the diagnosis efficiency and accuracy.

## Future work

With the development of NLP technology, especially the rise of large models such as ChatGPT, NLP will have a wider and deeper application in the field of ophthalmology. For example, ChatGPT can be used to provide accurate and high-quality information to answer questions about myopia [34]. It can also be used to optimize the writing process from introduction to organization and discussion, thereby accelerating the progress of ophthalmic research [35]. Choi J Y et al. pointed out that in the future, research teams that can effectively utilize large models and are good at fine-tuning them to adapt to their specific scientific research tasks will become the new leaders in the field of combination of ophthalmology and AI [36]. Therefore, in terms of NLP processing, we will consider using other high-performance NLP algorithm models such as Transformers and Attentive to continuously improve the accuracy of medical vocabulary NER, and expand the scope of ophthalmic medical vocabulary. We will promote the diagnostic intelligent decision support platform to practical clinical applications, assisting doctors in diagnosis and treatment activities. At the same time, we will also use NLP to process more unstructured data of medical records, such as ophthalmic imaging reports, pathological reports, and surgical medical records. Through AI technology, we will integrate various medical information data of patients, create a diagnosis and treatment plan decision-making platform, and help ophthalmologists quickly and accurately customize the best personalized diagnosis and treatment plan for different patients.

## Data Availability

The datasets used and/or analyzed during the current study are available from the corresponding author on reasonable request.
